# Direct training high-performance deep spiking neural networks: a review of theories and methods

**DOI:** 10.3389/fnins.2024.1383844

**Published:** 2024-07-31

**Authors:** Chenlin Zhou, Han Zhang, Liutao Yu, Yumin Ye, Zhaokun Zhou, Liwei Huang, Zhengyu Ma, Xiaopeng Fan, Huihui Zhou, Yonghong Tian

**Affiliations:** ^1^Peng Cheng Laboratory, Shenzhen, China; ^2^Faculty of Computing, Harbin Institute of Technology, Harbin, China; ^3^School of Electronic and Computer Engineering, Shenzhen Graduate School, Peking University, Shenzhen, China; ^4^National Key Laboratory for Multimedia Information Processing, School of Computer Science, Peking University, Beijing, China

**Keywords:** deep spiking neural network, direct training, transformer-based SNNs, residual connection, energy efficiency, high performance

## Abstract

Spiking neural networks (SNNs) offer a promising energy-efficient alternative to artificial neural networks (ANNs), in virtue of their high biological plausibility, rich spatial-temporal dynamics, and event-driven computation. The direct training algorithms based on the surrogate gradient method provide sufficient flexibility to design novel SNN architectures and explore the spatial-temporal dynamics of SNNs. According to previous studies, the performance of models is highly dependent on their sizes. Recently, direct training deep SNNs have achieved great progress on both neuromorphic datasets and large-scale static datasets. Notably, transformer-based SNNs show comparable performance with their ANN counterparts. In this paper, we provide a new perspective to summarize the theories and methods for training deep SNNs with high performance in a systematic and comprehensive way, including theory fundamentals, spiking neuron models, advanced SNN models and residual architectures, software frameworks and neuromorphic hardware, applications, and future trends.

## 1 Introduction

Regarded as the third generation of neural network (Maass, 1997), the brain-inspired spiking neural networks (SNNs) are potential competitors to traditional artificial neural networks (ANNs) in virtue of their high biological plausibility, and low power consumption when implemented on neuromorphic hardware (Roy et al., 2019). In particular, the utilization of binary spikes allows SNNs to adopt low-power accumulation (AC) instead of the traditional high-power multiply-accumulation (MAC), leading to significantly enhanced energy efficiency and making SNNs increasingly popular (Chen et al., 2023).

There are two mainstream pathways to obtain deep SNNs: ANN-to-SNN conversion and direct training through the surrogate gradient method. Firstly, in ANN-to-SNN conversion (Cao et al., 2015; Hunsberger and Eliasmith, 2015; Rueckauer et al., 2017; Bu et al., 2022; Meng et al., 2022; Wang Y. et al., 2022), a pre-trained ANN is converted to an SNN by replacing the ReLU activation layers with spiking neurons and adding scaling operations like weight normalization and threshold balancing. This conversion process suffers from long converting time steps, which causes high computational consumption in practice. In addition, the converted SNNs obtained in this way are constrained by the original ANNs' architecture and are hard to adapt to dynamic signal (DVS, DAVIS, ATIS data) processing. Thus, the direct exploration of the virtues of SNNs is limited in ANN-to-SNN conversion. Secondly, in the field of direct training, SNNs are unfolded over simulation time steps and trained with backpropagation through time (Lee et al., 2016; Shrestha and Orchard, 2018). Due to the non-differentiability of spiking neurons, the surrogate gradient method is employed for backpropagation (Neftci et al., 2019; Lee et al., 2020b; Fang et al., 2021a,b; Zhou Z. et al., 2023). On one hand, this direct training method can handle temporal data and also achieve decent performance on large-scale static datasets, with only a few time steps. On the other hand, it can provide sufficient flexibility for designing novel architectures specifically for SNNs and exploring the properties of SNNs directly. Therefore, the direct training method has received more attention recently.

Given the significant benefits and rapid advancement of directly trained deep SNNs, particularly the emergence of high-performance transformer-based SNNs, this review systematically and comprehensively summarizes the theories and methods for directly trained deep SNNs. Combining theory fundamentals, spiking neuron models, advanced SNN models and residual architectures, software frameworks and neuromorphic hardware, applications, and future trends, this article offers fresh perspectives into the field of SNNs. This review is structured as follows: Section 2 presents the evolution and recent advancements in spiking neuron models. Section 3 introduces the fundamental principles of spiking neural networks. Section 4 focuses on the most recent advanced SNN models and architectures, especially transformer-based SNNs. Section 5 concludes the software frameworks for training SNNs and the development of neuromorphic hardware. Section 6 summarizes the applications of deep SNNs. Finally, Section 7 points out future research trends and concludes this review.

## 2 Spiking neuron models

**Figure 1:**
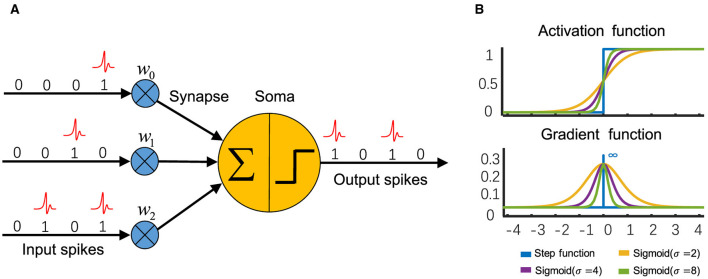
**(A)** The scheme of a spiking neuron, of which the input and output are both binary spikes. **(B)** The sigmoid function approximates the Heaviside activation function of a spiking neuron, and its derivative can be utilized to calculate gradients during backpropagation.

LIF (Leaky Integrate-and-Fire) neuron is one of the most commonly used neurons in SNNs (Zhou et al., 2023a,b; Zhou Z. et al., 2023), which is simple but retains biological characteristics (Figure 1A). The dynamics of LIF are described as Equations (1–3):


(1) $$\begin{array}{l} H\left[t\right]=V\left[t-1\right]+\frac{1}{\tau }\left(X\left[t\right]-\left(V\left[t-1\right]-V_{reset}\right)\right), \end{array}$$

(2) $$\begin{array}{l} S\left[t\right]=\Theta \left(H\left[t\right]-V_{th}\right), \end{array}$$

(3) $$\begin{array}{l} V\left[t\right]=H\left[t\right]\left(1-S\left[t\right]\right)+V_{reset}S\left[t\right], \end{array}$$

where τ in Equation (1) is the membrane time constant, *X*[*t*] is the input current at time step *t*. *V*_*reset*_ represents the reset potential, *V*_*th*_ represents the spike firing threshold, *H*[*t*] and *V*[*t*] represent the membrane potential before and after spike firing at time step *t*, respectively. Θ(*v*) is the Heaviside step function, if *v* ≥ 0 then Θ(*v*) = 1, meaning a spike is generated; otherwise Θ(*v*) = 0. *S*[*t*] represents whether a neuron fires a spike at time step *t*.

LIF also comes with notable limitations in practical applications. For instance, LIF needs to manually adjust the hyperparameters, such as membrane time constant τ and firing threshold *V*_*th*_, which constrains its expressiveness. In addition, LIF is simple in modeling, which limits the range of neuronal dynamics. Overall, there is a lack of diversity and flexibility in LIF, which calls for more advanced neuron models to enhance SNNs' performance and broaden their applications. Table 1 lists some recently developed spiking neuron models and their performance on typical tasks.

**Table 1:** Overview of spiking neurons for direct training and their performance.

**Method**	**Architecture**	**Dataset**	**Acc (%)**	**Training**
PLIF (Fang et al., 2021b)	PLIF-Net	CIFAR10	93.50	Time dependent
LTMD (Wang S. et al., 2022)	DenseNet	CIFAR10	94.19	Time dependent
GLIF (Yao et al., 2022)	ResNet-34	CIFAR10	95.03	Time dependent
MLF (Feng L. et al., 2022)	DS ResNet	CIFAR10	94.25	Time dependent
LIFB (Shen et al., 2023)	ResNet-19	CIFAR10	96.32	Time dependent
Deit-SNN (Rathi and Roy, 2023)	VGG16	CIFAR10	93.44	Time dependent
KLIF (Jiang and Zhang, 2023)	CNN	CIFAR10	92.52	Time dependent
MT-SNN (Wang X. et al., 2023)	MT-VGG9	CIFAR10	94.74	Time dependent
PSN (Fang et al., 2023b)	PLIF-Net	CIFAR10	95.32	Parallel
GLIF (Yao et al., 2022)	ResNet-34	CIFAR100	77.35	Time dependent
Deit-SNN (Rathi and Roy, 2023)	VGG16	CIFAR100	69.67	Time dependent
LIFB (Shen et al., 2023)	ResNet-19	CIFAR100	78.31	Time dependent
MT-SNN (Wang X. et al., 2023)	MT-VGG9	CIFAR100	75.53	Time dependent
GLIF (Yao et al., 2022)	ResNet-34	ImageNet	69.09	Time dependent
Deit-SNN (Rathi and Roy, 2023)	VGG16	ImageNet	69.00	Time dependent
LIFB (Shen et al., 2023)	SEW ResNet-34	ImageNet	70.02	Time dependent
PSN (Fang et al., 2023b)	SEW ResNet-34	ImageNet	70.54	Parallel
PLIF (Fang et al., 2021b)	PLIF-Net	CIFAR10-DVS	74.80	Time dependent
GLIF (Yao et al., 2022)	ResNet-34	CIFAR10-DVS	78.10	Time dependent
MLF (Feng L. et al., 2022)	DS ResNet	CIFAR10-DVS	70.36	Time dependent
LTMD (Wang S. et al., 2022)	DenseNet	CIFAR10-DVS	73.30	Time dependent
KLIF (Jiang and Zhang, 2023)	CNN	CIFAR10-DVS	70.90	Time dependent
MT-SNN (Wang X. et al., 2023)	MT-VGG9	CIFAR10-DVS	76.30	Time dependent
PSN (Fang et al., 2023b)	VGG	CIFAR10-DVS	85.90	Parallel
PLIF (Fang et al., 2021b)	PLIF-Net	DVS128-Gesture	97.57	Time dependent
MLF (Feng L. et al., 2022)	DS ResNet	DVS128-Gesture	97.29	Time dependent
KLIF (Jiang and Zhang, 2023)	CNN	DVS128-Gesture	94.10	Time dependent
LSNN (Bellec et al., 2018)	LSTM	Sequential	96.40	Time dependent
		MNIST		
ASN (Yin et al., 2020)	RNN	PS-MNIST	97.90	Time dependent
SPSN (Yarga and Wood, 2023)	MLP	SHD	86.89	Parallel

### 2.1 Spiking neurons with trainable parameters

Based on LIF, many improved spiking neuron models with trainable parameters have been proposed, which expand the representation space of neurons through parameter learning and improve the expression ability of SNNs. Fang et al. proposed Parametric LIF (PLIF) (Fang et al., 2021b) by using trainable membrane time constant as follows:


(4) $$\begin{array}{l} H\left[t\right]=V\left[t-1\right]+k\left(a\right)\left(X\left[t\right]-\left(V\left[t-1\right]-V_{reset}\right)\right), \end{array}$$

where *k*(*a*) in Equation (4) denotes a clamp function and $k\left(a\right)=\frac{1}{1+exp\left(-a\right)}\in \left(0,1\right)$. The trainable membrane-related parameter of PLIF is biologically plausible, as neurons in the brain are heterogeneous. LTMD (Wang S. et al., 2022) also leverages this biological plausibility but approaches it differently by employing learnable firing thresholds. An increase in the threshold of LTMD results in a reduction of output spikes, making an SNN less sensitive to its input and thus more robust. On the contrary, a decrease in the threshold leads to an increment of output spikes, making an SNN more sensitive to its input, which is particularly beneficial for processing transient small signals. Therefore, the learnable threshold *V*_*th*_ = tank(*k*), of which *k* is trainable, can lead to the optimal sensitivity of an SNN.

Diet-SNN (Rathi and Roy, 2023) adopts an end-to-end gradient descent optimization algorithm to train the membrane-related parameters and firing thresholds of LIF neurons while optimizing the network weights. The trained neuron parameters selectively reduce the membrane potential, making spikes in the network sparser, thereby improving the computational efficiency of SNN. Spiking neurons with dynamic thresholds are adopted in LSNN (Bellec et al., 2018). After firing a spike each time, the firing threshold of a neuron will increase by a fixed amount, and then it will decay exponentially according to the time constant. Adaptive spiking neuron (ASN) (Yin et al., 2020) was proposed for sequence and streaming media tasks. In ASN, the time constant of membrane potential is trainable. In addition, similar to LSNN, the firing threshold will increase after each spike of the neuron, thus improving sparsity and efficiency.

In KLIF (Jiang and Zhang, 2023), a trainable scaling factor *k* and a nonlinear ReLU activation function are inserted between charging and firing. The dynamics of KLIF can be described by Equations (1), (5–7).


(5) $$\begin{array}{l} F\left[t\right]=\text{ReLU}\left(kH\left[t\right]\right), \end{array}$$

(6) $$\begin{array}{l} S\left[t\right]=\Theta \left(F\left[t\right]-V_{th}\right), \end{array}$$

(7) $$\begin{array}{l} V\left[t\right]=F\left[t\right]\left(1-S\left[t\right]\right)+V_{reset}S\left[t\right]. \end{array}$$

Compared with LIF, KLIF can automatically adjust the membrane potential and the gradient of backpropagation within the neuron. GLIF (Yao et al., 2022) introduces a gating unit that fuses multiple biometric features, with the ratio of these features adjusted by a trainable gating factor. Moreover, inspired by various spiking patterns of brain neurons, LIFB (Shen et al., 2023) has three modes: resting, regular spiking, and burst spiking. The density of the burst spiking can be learned automatically, which greatly enriches the representation capability of neurons.

In addition, there are other studies trying to improve performance by multi-level firing thresholds instead of trainable parameters. To reduce the performance loss caused by the transmission of binarized spikes in the network, MT-SNN (Wang X. et al., 2023) introduces multi-level firing thresholds. MT-SNN performs convolution operations on the binarized spikes generated by different firing thresholds and then sums them up. Similarly, MLF (Feng L. et al., 2022) can also fire spikes under different firing thresholds, thus improving the performance of SNNs.

### 2.2 Parallel spiking neurons

A typical neuron model like LIF is time-dependent, that is, its state at time *t* relies on its state at time *t* − 1, resulting in a high computation load. Fang et al. (2023b) proposed a parallel spiking neuron (PSN) to accelerate the computation by parallel computing. By eliminating the resetting process, they represent the charging process of PSN by a non-iterative equation as Equation (8):


(8) $$\begin{array}{l} H\left[t\right]=\overset{T-1}{\underset{i=0}{\sum }}W_{t,i}\cdot X\left[i\right], \end{array}$$

where *W*_*t,i*_ is the weight between input *X*[*i*] and membrane potential *H*[*t*]. For LIF neuron, $W_{t,i}=\frac{1}{\tau_{m}}{\left(1-\frac{1}{\tau_{m}}\right)}^{t-i}$. The dynamics of PSN are as Equations (9, 10):


(9) $$\begin{array}{l} H=WX,\;W\in {\mathbb{R}}^{T\times T},X\in {\mathbb{R}}^{T\times N} \end{array}$$

(10) $$\begin{array}{l} S=\Theta \left(H-B\right),\;B\in {\mathbb{R}}^{T},S\in {\left\{0,1\right\}}^{T\times N} \end{array}$$

where ***X*** is the input, ***W*** and ***B*** are trainable weights and trainable firing thresholds, respectively. ***H*** is the membrane potential after charging, and ***S*** denotes whether a neuron spikes. *N* and *T* are the batch size and the number of time steps, respectively. For step-by-step serial forward computation and variable-length sequence processing, the masked PSN and the sliding PSN are also derived.

The stochastic parallel spiking neuron (SPSN) (Yarga and Wood, 2023) adopts an idea similar to PSN, by removing the resetting mechanism. The neuronal dynamics of SPSN contains two parts, namely parallel leaky integrator and stochastic firing. The leaky integrator is a linear time-invariant system, which can be transformed into the Fourier domain to realize parallel computation. Stochastic firing adaptively adjusts the firing probability through trainable parameters, enhancing the network's capability to process information in a dynamic and efficient manner.

## 3 Fundamentals of spiking neural networks

### 3.1 Information coding

To process image data through SNNs, it is essential to first encode the data into spike trains. Rate coding (Adrian and Zotterman, 1926) is the most commonly used information coding method in SNNs, in which the firing rate is proportional to the intensity of the input signal and spikes are typically generated by a Poisson process (Wiener and Richmond, 2003). To encode information more accurately, rate coding requires a longer time window, which leads to a slower information transmission rate. In contrast, utilizing a shorter time window may result in loss of information during encoding, presenting a trade-off between speed and accuracy in information transmission.

Different from rate coding, temporal coding represents information through the timing of spikes. Time-to-first-spike (TTFS) (Park et al., 2020; Guo W. et al., 2021) stands out for its simplicity and efficiency in temporal coding, which uses the time of the first spike fired by the neuron to represent the input signal. TTFS effectively reduces the total number of spikes, thereby accelerating the computation of SNNs. TTFS algorithm can be described as Equation (11):


(11) $$\begin{array}{l} S\left[t\right]=\{\begin{array}{cc} 1, & \text{if}t=\left(\frac{X_{max}-X}{X_{max}}\right)t_{max}\\ 0, & \text{otherwise} \end{array}\;, \end{array}$$

where *S*[*t*] represents whether a spike is fired at time *t* after encoding, *t*_*max*_ denotes the maximum time allowed during encoding, *X* and *X*_*max*_ represent the input signal and its maximum value, respectively. In the TTFS encoding method, larger values of the input signal lead to earlier firing of spikes.

### 3.2 Network training

#### 3.2.1 Surrogate gradient

As the core components of SNNs, neurons are essential for information processing and transmission, since spikes are fired by neurons. However, the firing of spikes involves the non-differentiable Heaviside step function, which presents a significant challenge in the direct training of SNNs. To address the non-differentiability of the Heaviside step function, Neftci et al. (2019) proposed the Surrogate Gradient (SG) algorithm. In SG, the Heaviside step function is adopted to generate spikes during forward propagation, and differentiable functions are adopted for gradient calculation during backpropagation. Notably, SG functions could vary according to the networks. For instance, the SG function used in SEW ResNet (Fang et al., 2021a) is the derivative of the arctan function as follows:


(12) $$\begin{array}{l} \sigma \left(x\right)=\frac{1}{\pi }\arctan\left(\frac{\pi }{2}\alpha x\right)+\frac{1}{2}\;, \end{array}$$

(13) $$\begin{array}{l} \sigma^{\prime }\left(x\right)=\frac{\alpha }{2\left(1+{\left(\frac{\pi }{2}\alpha x\right)}^{2}\right)}\;. \end{array}$$

Equation (13) is the derivative of Equation (12). In addition, SG could be the derivative of Sigmoid (Figure 1B) (Zhou et al., 2023a,b; Zhou Z. et al., 2023), tanh (Guo et al., 2022a), or rectangular (Wu et al., 2018, 2019) functions, etc. To address the problem of gradient vanishing caused by a surrogate gradient function with fixed parameters, Lian et al. (2023) proposed the Learnable Surrogate Gradient (LSG), in which a learnable parameter is used to adjust the gradient-available interval.

Li et al. (2021) proposed Differentiable Spike (Dspike) as another approach to overcome the non-differentiable problem of the Heaviside function. Based on the hyperbolic tangent function, Dspike can be described as Equation (14):


(14) $$\begin{array}{l} Dspike\left(x,b\right)=\frac{\tanh\left(b\left(x-0.5\right)\right)+\tanh\left(b/2\right)}{2\left(\tanh\left(b/2\right)\right)},\text{if }0\leq x\leq 1 \end{array}$$

By adjusting the parameter *b*, different backpropagation gradients can be obtained. Differentiation on Spike Representation (DSR) proposed by Meng et al. (2022) encodes spike trains and represents them as sub-differentiable mapping, which also avoids the non-differentiable problem during backpropagation.

#### 3.2.2 Loss function and backpropagation

Loss function is the key to neural network training, and different loss functions have been proposed to enhance the performance of SNNs. IM-Loss (Guo et al., 2022a), for example, aims to maximize the information flow in the network. The total loss function consists of two parts, cross-entropy loss, and IM-Loss, as Equations (15, 16):


(15) $$\begin{array}{l} L_{Total}=L_{CE}+\lambda L_{IM}, \end{array}$$

(16) $$\begin{array}{l} L_{IM}=\overset{L}{\underset{l=0}{\sum }}{\left({Ū}_{l}-V_{th}\right)}^{2}/L, \end{array}$$

where Ū_*l*_ is the averaged membrane potential at all time steps of the *l*-th layer, and *L* is the total number of layers. To alleviate the information loss in SNNs and reduce the quantization error, RMP-Loss (Guo et al., 2023a) is proposed to adjust the distribution of membrane potential. RecDis-SNN (Guo et al., 2022b) adopts MDP-Loss that also adjusts the membrane potential distribution to overcome the distribution shift during network training. In addition, to improve the generalization ability of SNNs, Deng et al. proposed temporal efficient training (TET) (Deng et al., 2022) loss function to make the network output closer to the target distribution.

**Figure 2:**
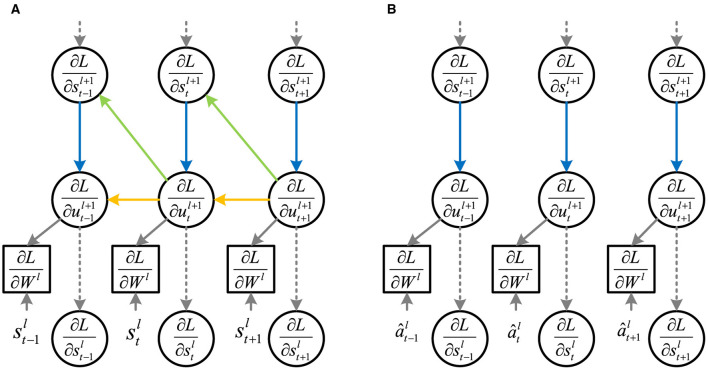
The backward of **(A)** BPTT and **(B)** OTTT.

Distinct from ANNs, there's an additional dimension in SNNs, the temporal domain. For spiking neurons, the membrane potential in the current step depends on the membrane potential in the previous time step, that is, there is a time dependence. Thus, backpropagation in ANNs does not apply to SNNs. Backpropagation Through Time (BPTT) (Werbos, 1990; Bird and Polivoda, 2021), originally developed for recurrent neural networks (RNNs), is applied to SNNs due to their similar characteristics to those of RNNs. The combination of BPTT and surrogate gradient is the basic approach in SNNs. Spatio-temporal backpropagation (STBP) (Wu et al., 2018), proposed by Wu et al., takes the gradient update in both the spatial domain and temporal domain into account to train SNNs. However, the additional time dimension exposes BPTT and STBP to the problem of requiring a large amount of training memory and training time. Therefore, Xiao et al. (2022) proposed an online training through time (OTTT) algorithm derived from BPTT, which only requires constant training memory consumption agnostic to time steps, and reduces the significant memory costs compared to BPTT. The backward of BPTT and OTTT are shown in Figure 2. Another efficient backpropagation method, Spatial Learning Through Time (SLTT) (Meng et al., 2023), ignores the unimportant routes in the computational graph during backpropagation, to reduce training memory consumption and training time. However, although OTTT and SLTT show better training memory consumption than BPTT, direct training high-performance SNNs are still dominated by the combination of BPTT and surrogate gradient, such as SGLFormer (Zhang et al., 2024), Spikformer (Zhou Z. et al., 2023), etc. Thus, it's essential to investigate direct training methods offering both high effectiveness and efficiency.

#### 3.2.3 Batch normalization

In SNNs, batch normalization is an indispensable component, especially in the context that deep SNNs are difficult to train and converge, compared to ANNs. To mitigate the degradation problems of SNNs, Zheng et al. (2021) proposed threshold-dependent batch normalization (tdBN), which is described as Equation (17):


(17) $$\begin{array}{l} {\hat{X}}_{k}=\gamma_{k}\frac{\alpha V_{th}\left(X_{k}-\mu \right)}{\sqrt{\sigma^{2}+\epsilon }}+\beta_{k}, \end{array}$$

where α is a hyperparameter, *V*_*th*_ is the firing threshold of the neuron, *X*_*k*_ is the feature of the *k*-th channel, γ_*k*_ and β_*k*_ are trainable parameters, μ and σ^2^ are mean and variance, respectively, ϵ is a tiny constant. Temporal effective batch normalization (TEBN) (Duan et al., 2022) regularizes the temporal distribution, by adopting batch normalization with different parameters at different time steps. Batch normalization through time (BNTT) proposed by Kim and Panda (2021) is similar to TEBN, which also adopts different batch normalization parameters for feature maps at different time steps. Moreover, Guo et al. (2023b) applied batch normalization inside the LIF neuron to normalize the distribution of membrane potentials before firing spikes.

## 4 SNN architecture developments

This review focuses on the most recent SNN models. Recently, the evolution of residual blocks enhances both the size and performance of deep SNNs significantly. In addition, combining SNNs with transformer architecture has broken the bottleneck of SNNs' performance. Therefore, this review focuses on the application of two kinds of architectures in direct training deep SNNs: transformer structures (Section 4.1) and the residual connections (Section 4.2). Table 2 summarizes their performance on mainstream datasets (ImageNet-1K, CIFAR10, CIFAR100, DVS128 Gesture, CIFAR10-DVS).

**Table 2:** Overview of direct training deep SNNs and their performance on ImageNet, CIFAR10, CIFAR100, DVS128-Gesture, CIFAR10-DVS.

**Method**	**Architecture**	**Param (M)**	**Time steps**	**Dataset**	**Top-1 Acc (*%*)**
Spiking ResNet (Hu et al., 2021a)	ResNet-50	25.56	350	ImageNet	72.75
SEW ResNet (Fang et al., 2021a)	SEW-ResNet-152	60.19	4	ImageNet	69.26
MS-ResNet (Hu et al., 2021b)	MS-ResNet-104	77.28	5	ImageNet	76.02
Att MS-ResNet (Yao et al., 2023b)	Att-MS-ResNet-104	78.37	4	ImageNet	77.08
Spikformer (Zhou Z. et al., 2023)	Spikformer-8-768	66.34	4	ImageNet	74.81
Spikingformer (Zhou et al., 2023a)	Spikingformer-8-768	66.34	4	ImageNet	75.85
CML (Zhou et al., 2023b)	Spikformer-8-768	66.34	4	ImageNet	77.34
Spike-driven Transformer (Yao et al., 2023a)	S-Transformer-8-768	66.34	4	ImageNet	77.07
SpikingResformer (Shi et al., 2024)	SpikingResformer-L	60.38	4	ImageNet	79.40
Spike-driven Transformer V2 (Yao et al., 2024)	Meta-SpikeFormer	55.40	4	ImageNet	80.00
Spikformer V2 (Zhou Z. et al., 2024)	Spikformer V2-8-512	51.55	4	ImageNet	80.38
SGLFormer (Zhang et al., 2024)	SGLFormer-8-768	64.02	4	ImageNet	83.73
QKFormer (Zhou C. et al., 2024)	HST-10-768	64.96	4	ImageNet	85.65
Hybrid training (Rathi et al., 2020)	VGG-11	9.27	125	CIFAR10	92.22
STBP-tdBN (Zheng et al., 2021)	ResNet-19	12.63	4	CIFAR10	92.92
TET (Deng et al., 2022)	ResNet-19	12.63	4	CIFAR10	94.44
MS-ResNet (Hu et al., 2021b)	MS-ResNet-110	–	4	CIFAR10	92.12
Spikformer (Zhou Z. et al., 2023)	Spikformer-4-384	9.32	4	CIFAR10	95.51
Spikingformer (Zhou et al., 2023a)	Spikingformer-4-384	9.32	4	CIFAR10	95.81
CML (Zhou et al., 2023b)	Spikformer-4-384	9.32	4	CIFAR10	96.04
Spike-driven Transformer (Yao et al., 2023a)	S-Transformer-2-512	10.23	4	CIFAR10	95.60
SGLFormer (Zhang et al., 2024)	SGLFormer-4-384	8.85	4	CIFAR10	96.76
Hybrid training (Rathi et al., 2020)	VGG-11	9.27	125	CIFAR100	67.87
STBP-tdBN (Zheng et al., 2021)	ResNet-19	12.63	4	CIFAR100	70.86
TET (Deng et al., 2022)	ResNet-19	12.63	4	CIFAR100	74.47
Spikformer (Zhou Z. et al., 2023)	Spikformer-4-384	9.32	4	CIFAR100	78.21
Spikingformer (Zhou et al., 2023a)	Spikingformer-4-384	9.32	4	CIFAR100	79.21
CML (Zhou et al., 2023b)	Spikformer-4-384	9.32	4	CIFAR100	80.02
Spike-driven Transformer (Yao et al., 2023a)	S-Transformer-2-512	10.28	4	CIFAR100	78.4
SGLFormer (Zhang et al., 2024)	SGLFormer-4-384	8.88	4	CIFAR100	82.26
SEW-ResNet (Hu et al., 2021b)	SEW-ResNet	–	16	DVS128-Gesture	97.9
tdBN (Zheng et al., 2021)	ResNet	–	40	DVS128-Gesture	96.9
Spikformer (Zhou Z. et al., 2023)	Spikformer-2-256	2.57	16	DVS128-Gesture	98.3
Spikingformer (Zhou et al., 2023a)	Spikingformer-2-256	2.57	16	DVS128-Gesture	98.3
CML (Zhou et al., 2023b)	Spikformer-2-256	2.57	16	DVS128-Gesture	98.6
Spike-driven Transformer (Yao et al., 2023a)	S-Transformer-2-256	2.57	16	DVS128-Gesture	99.3
STSA (Wang Y. et al., 2023)	STSFormer-2-256	1.99	16	DVS128-Gesture	98.72
SGLFormer (Zhang et al., 2024)	SGLFormer-3-256	2.17	16	DVS128-Gesture	98.6
SEW-ResNet (Hu et al., 2021b)	SEW-ResNet	–	16	CIFAR10-DVS	74.4
Spikformer (Zhou Z. et al., 2023)	Spikformer-2-256	2.57	16	CIFAR10-DVS	80.9
Spikingformer (Zhou et al., 2023a)	Spikingformer-2-256	2.57	16	CIFAR10-DVS	81.3
CML (Zhou et al., 2023b)	Spikformer-2-256	2.57	16	CIFAR10-DVS	80.9
Spike-driven Transformer (Yao et al., 2023a)	S-Transformer-2-256	2.57	16	CIFAR10-DVS	80.0
STSA (Wang Y. et al., 2023)	STSFormer-2-256	1.99	16	CIFAR10-DVS	79.93
SGLFormer (Zhang et al., 2024)	SGLFormer-3-256	2.58	10	CIFAR10-DVS	82.9

### 4.1 Transformer-based spiking neural networks

Transformer, originally designed for natural language processing (Vaswani et al., 2017), has achieved great success in many computer vision tasks, including image classification (Dosovitskiy et al., 2021; Yuan et al., 2021), object detection (Carion et al., 2020; Liu et al., 2021; Zhu X. et al., 2021), and semantic segmentation (Wang et al., 2021; Yuan et al., 2022). While convolution-based models mainly rely on inductive bias and focus on adjacent pixels, transformer structures use self-attention to capture the relation among spiking features globally, which enhances the performance effectively.

**Figure 3:**
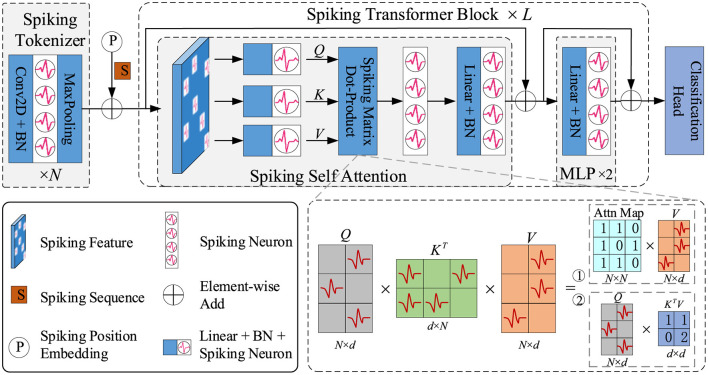
The overview of spiking transformer (Spikformer).

To adopt transformer structure in SNNs, Zhou Z. et al. (2023) designed a novel spike-form self-attention named Spiking Self Attention (SSA), using sparse spike-form Query, Key and Value without softmax operation. The calculation process of SSA is formulated as Equations (18–20):


(18) $$\begin{array}{l} Q=\text{S}{\text{N}}_{Q}\left(\text{BN}\left(XW_{Q}\right)\right),K=\text{S}{\text{N}}_{K}\left(\text{BN}\left(XW_{K}\right)\right),\\ V=\text{S}{\text{N}}_{V}\left(\text{BN}\left(XW_{V}\right)\right), \end{array}$$

(19) $$\begin{array}{l} {\mathrm{SSA}}^{\prime }\left(Q,K,V\right)=\text{SN}\left(QK^{\text{T}}V*s\right), \end{array}$$

(20) $$\begin{array}{l} \mathrm{SSA}\left(Q,K,V\right)=\text{SN}\left(\mathrm{BN}\left(\mathrm{Linear}\left({\mathrm{SSA}}^{\prime }\left(Q,K,V\right)\right)\right)\right), \end{array}$$

where *Q, K, V* ∈ ℝ^*T*×*N*×*D*^. The spike-form Query (*Q*), Key (*K*), and Value (*V*) are computed by learnable layers. *s* is a scaling factor, which can be fused into the next spiking neuron in practice. Therefore, the calculation of SSA avoids multiplication, meeting the property of SNNs. Based on the SSA, Zhou Z. et al. (2023) developed a spiking transformer named Spikformer, which is shown in Figure 3. As the first transformer-based SNN model, Spikformer achieves 74% accuracy on ImageNet-1k, showing great performance potential.

Zhou et al. (2023a) discussed the non-spike computation problem (integer-float multiplications) of Spikformer (Zhou Z. et al., 2023) and SEW-ResNet (Fang et al., 2021a), which is caused by Activation-after-addition shortcut. Spikingformer (Zhou et al., 2023a) was proposed with the Pre-activation shortcut to avoid the non-spike computation problem in synaptic computing. Experimental Analysis has shown that Spikingformer has only about 43% energy consumption compared with Spikformer in synaptic computing, with only accumulation operations and lower fire rates. CML (Zhou et al., 2023b) designed a downsampling structure specifically for SNNs to solve the imprecise gradient backpropagation problem of most state-of-the-art deep SNNs (including Spikformer). CML achieved 77.34% on ImageNet, significantly enhancing the performance of transformer-based SNNs. All the architectures above are based on SSA with computational complexity of *O*(*N*^2^*d*) or *O*(*Nd*^2^), while Yao et al. (2023a) designed a novel Spike-Driven Self-Attention (SDSA) with linear complexity regarding both the number of tokens and channels. SDSA uses only mask and addition operations without any multiplication, thus having up to 87.2 × lower computation energy than the vanilla SSA. In addition, the Spike-driven Transformer based on SDSA has achieved 77.1% accuracy on ImageNet-1k. Wang Y. et al. (2023) proposed an SNN-based spatial-temporal self-attention (STSA) mechanism, which could calculate the feature dependence across the time and space domains. Shi et al. (2024) proposed Dual Spike Self-Attention (DSSA) with a reasonable scaling method, achieving 79.40% top-1 accuracy on ImageNet-1K. Yao et al. (2024) proposed Spike-driven Transformer v2 which explored the impact of structure, spike-driven self-attention, and skip connection on its performance to inspire the next-generation transformer-based neuromorphic chip designs. Zhou Z. et al. (2024) developed a Spiking Convolutional Stem (SCS) with supplementary layers to enhance the architecture of Spikformer, achieving 80.38% accuracy on ImageNet-1k. Zhang et al. (2024) proposed a Spiking Global-Local-Fusion Transformer (SGLFormer), which enables efficient information processing on both global and local scales, by integrating transformer and convolution structures in SNNs. SGLFormer achieved a groundbreaking top-1 accuracy of 83.73% on ImageNet-1k with 64M parameters. Zhou C. et al. (2024) proposed QKFormer, a novel hierarchical spiking transformer using Q-K attention, which can easily model the importance of token or channel dimensions with binary values and has linear complexity to #tokens (or #channels). QKFormer achieved a significant milestone, surpassing 85% top-1 accuracy on ImageNet with 4 time steps using the direct training approach.

Biological realistic models tend to model neural networks with high biological plausibility to simulate the complex biological mechanism of the brain. It often lacks the consideration of computational efficiency and performance optimization on general application tasks. Traditional ANNs often prioritize task performance over biological realism and computational energy consumption. SNNs have great potential to own the characteristics of biological plausibility, low computational energy consumption, and high task performance simultaneously. Especially, several direct training Transformer-based SNNs have broken through 80% top-1 accuracy on ImageNet-1K, which instills great optimism in the application of SNNs.

### 4.2 Residual architectures in spiking neural networks

**Figure 4:**
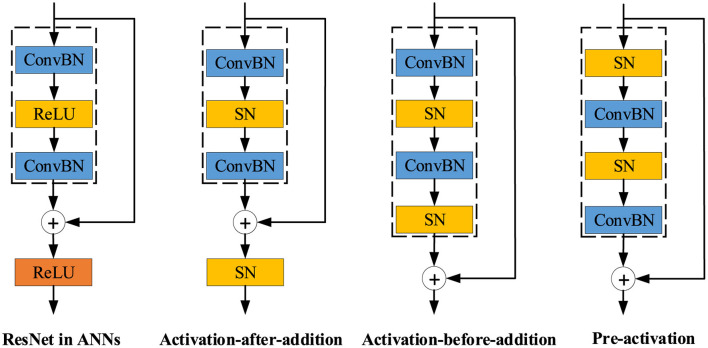
The overview of residual learning architectures.

Residual block is the fundamental block in both deep ANNs and SNNs. As shown in Figure 4, there are mainly three residual shortcut types in SNNs: Activation-after-addition, Activation-before-addition, and Pre-activation. Both advantages and disadvantages of these three types are concluded in Table 3. **Activation-after-addition shortcut** simply replaces ReLU activation layers in the standard residual block with spiking neurons, such as Spiking ResNet (Hu et al., 2021a) and MPBN (Guo et al., 2023b). SNNs with this simple design suffer from performance degradation and gradient vanishing/exploding. For example, the deeper 34-layer Spiking ResNet has lower test accuracy than the shallower 18-layer Spiking ResNet. As the layer increases, the test accuracy of Spiking ResNet decreases (Fang et al., 2021a). To solve the degradation problem in the Activation-after-addition shortcut, **Activation-before-addition shortcut** is proposed in SEW-ResNet (Fang et al., 2021a), which extended directly trained SNNs to 100 layers for the first time. This structure has been widely used, such as in Spikformer (Zhou Z. et al., 2023), PLIF (Fang et al., 2021b), PSN (Fang et al., 2023b). This design mitigates the vanishing/exploding gradient problem and could train deeper SNN. However, the blocks in this shortcut will result in positive integers, which leads to non-spike computations (integer-float multiplications) in synaptic computing (like convolutional layer, linear layer) (Zhou et al., 2023a). **Pre-activation shortcut** could be traced back to the *Activation*-*Conv*-*Bn* paradigm, which is a fundamental building block in Binary Neural Networks (BNNs) (Liu et al., 2018, 2020; Guo N. et al., 2021; Zhang Y. et al., 2022). Some representative SNNs that use the Pre-activation shortcut include MS-ResNet (Hu et al., 2021b), Spikingformer (Zhou et al., 2023a), Spike-driven transformer (Yao et al., 2023a). MS-ResNet directly trained convolution-based SNNs to successfully extend the depth up to 482 layers on CIFAR10 without experiencing degradation problems, effectively verifying the feasibility of this way. Spikingformer (Zhou et al., 2023a) showed that the Pre-activation shortcut can effectively avoid non-spike computations, and thus has lower energy consumption than the previous shortcut in synaptic computing, through avoiding integer-float multiplication problems and with a lower firing rate. However, the Pre-activation shortcut requires dense transmission of floats in the residual branch.

**Table 3:** Features of various residual learning architectures.

**Features**	**Activation-after-addition**	**Activation-before-addition**	**Pre-activation**
Element addition	Spike added to floats	Integer added to spike	Floats added to floats
Gradient vanishing/exploding	Yes	No	No
Synaptic computing type	Spike computing	Multiplication between sparse integer and floats	Spike computing
Data transmission in residual branch	Spike	Sparse integer	Floats

Overall, the residual learning suitable for the properties of SNNs needs further exploration. In our opinion, the Activation-after-addition shortcut with gradient problem is not suitable for directly training deep SNNs, but is feasible in the field of ANN-to-SNN conversion. Activation-before-addition shortcut has some alternatives to ensure the properties of SNNs by slightly sacrificing the performance, such as using AND or IAND to replace ADD in the aggregation operation. Pre-activation shortcut needs further analyses of the effects of float transmission, and more efforts to exploit its advantages through collaborative hardware optimization and design.

### 4.3 Others

Besides the above-mentioned architectures, some other interesting research topics are also worthy of attention, such as Spiking RNN/LSTM, LSM, etc. Deep Liquid State Machine (LSM) (Wang and Li, 2016) explored the power of recurrent spiking networks and deep architectures. Soures and Kudithipudi (2019) proposed a novel deep LSM to capture dynamic information over multiple time-scales with a combination of randomly connected layers and unsupervised layers. Hamilton et al. (2019) demonstrated the nonlinear dynamics of spiking neurons can be used to implement low-level graph operations. Zhu Z. et al. (2022) proposed end-to-end Spiking Graph Convolutional Networks (GCNs) that integrate the embedding of GCNs with the biofidelity characteristics of SNNs. Bellec et al. (2020) and Bohnstingl et al. (2022) explored the architectures and online-training methods of recurrent spiking neural networks. Ren H. et al. (2023) proposed a novel end-to-end point-based SNN architecture, which excels at processing sparse event cloud data, effectively extracting both global and local features through a singular-stage structure.

## 5 Software frameworks and neuromorphic hardware for spiking neural networks

### 5.1 Software frameworks for training spiking neural networks

Software frameworks play a crucial role in propelling the advancement of deep learning. Deep learning frameworks such as PyTorch (Paszke et al., 2019) and TensorFlow (Abadi et al., 2016) leverage low-level languages like C++ libraries for high-performance acceleration on the backend, while offering user-friendly front-end application programming interfaces (APIs) implemented in high-level languages like Python. These frameworks significantly ease the workload of constructing and training ANNs, making substantial contributions to the growth of deep learning research. However, these deep learning frameworks are primarily designed for ANNs. With the development of large-scale brain-inspired neural networks, many related frameworks have emerged, facilitating the modeling and efficient computation of large-scale SNNs.

One category of frameworks includes brain simulators such as NEURON (Hines and Carnevale, 1997) and Brian (Goodman and Brette, 2009), which not only enhance the scalability and computational efficiency of models but also encompass cognitive functions such as perception, decision-making, and reasoning. The SNNs constructed by these frameworks exhibit a high degree of biological plausibility, making them suitable for studying the functionalities of real neural systems. They support biologically interpretable learning rules such as Spike-Timing-Dependent Plasticity (STDP) (Bi and Poo, 1998), playing a significant role in advancing the field of neuroscience. However, these frameworks lack core computational functionalities required for deep learning, such as automatic differentiation, rendering them incapable of performing machine learning tasks.

Another category of brain-inspired computing frameworks comprises deep spiking computation frameworks. Deep SNNs involve a substantial amount of matrix operations across spatial and temporal dimensions, a variety of neurons, neuromorphic datasets, and deployments on neuromorphic chips. The modeling and application processes are complex, and achieving high-performance acceleration is challenging. To address these issues, spiking deep learning frameworks need to support the construction, training, and deployment of deep SNNs, and be capable of acceleration based on spike operations. Frameworks such as BindsNET (Hazan et al., 2018), NengoDL (Rasmussen, 2019), SpykeTorch (Mozafari et al., 2019), Norse, SpyTorch, SNNTorch (Eshraghian et al., 2023), and SpikingJelly (Fang et al., 2023a) have been developed. They utilize simple spiking neurons to reduce computational complexity, making them suitable for machine learning research. Among them, BindsNet (Hazan et al., 2018) primarily focuses on machine learning and reinforcement learning; NengoDL (Rasmussen, 2019) converts ANNs to obtain deep SNNs but does not support direct training of SNNs using surrogate gradient methods; SpyTorch is a demonstrative framework that only provides basic surrogate gradient examples; SpyTorch (Mozafari et al., 2019) introduces a new type of surrogate gradient method named SuperSpike. These frameworks can implement some simple machine learning and reinforcement learning models, but they still lack deep learning capabilities for SNNs. Norse is attempting to introduce the sparse and event-driven characteristics of SNNs and supports many typical spiking neuron models. It is in the development stage and has not been officially released yet. SNNTorch supports some variants of online backpropagation algorithms that are more biologically plausible and support large-scale SNN computation. SpikingJelly (Fang et al., 2023a) is a full-stack toolkit for preprocessing neuromorphic datasets, building deep SNNs, optimizing their parameters, and deploying SNNs on neuromorphic chips, which shows remarkable extensibility and flexibility, enabling users to accelerate custom models at low costs through multilevel inheritance and semiautomatic code generation. In summary, the development of existing software frameworks is essentially in its early stages, and there is still a long way to go in terms of functionality enhancement and performance optimization.

### 5.2 Neuromorphic hardware for spiking neural networks

Neuromorphic hardware provides computational power for neural network models, playing a crucial role in large-scale brain-like neural networks. Efficient hardware can significantly accelerate the training, evaluation, iteration, and real-world applications of large-scale brain-like models. In comparison to general-purpose processors, deep learning chips and brain-like chips are specialized chips that focus on the computational efficiency of deep learning tasks and brain-like computing tasks, aiming to achieve better power/performance/area ratios. Current deep learning chips, like general CPUs, are based on the Von Neumann architecture, with separate computing and storage units. Brain-like chips enhance computational efficiency by designing efficient storage and computation hierarchy, enabling parallel data flow and efficient reuse, thus improving computational efficiency. From an architectural perspective, current brain-like chips can be mainly divided into two categories: analog-digital hybrid circuits and fully digital circuits (Table 4).

**Table 4:** Overview of typical neuromorphic hardware.

**Chip**	**Developer**	**Network**	**Function**	**Arch**	**Scale**
**Hybrid digital-analog**					
BrainScaleS (Schemmel et al., 2010)	Heidelberg Uni	SNNs	Training	NMA	Large
Neurogrid (Benjamin et al., 2014)	Stanford	SNNs	Inference	NMA	Large
ROLLS (Qiao et al., 2015)	UZH	SNNs	Training	NMA	Small
DYNAPs (Moradi et al., 2017)	UZH	SNNs	Inference	NMA	Small
Memristor-based (Zhang et al., 2021)	–	ANNs/SNNs	Inference	CIM	Small
BrainScaleS-2 (Pehle et al., 2022)	Heidelberg Uni	ANNs/SNNs	Training	NMA	Large
**Digital**					
SpiNNaker (Painkras et al., 2013)	UoM	SNNs	Training	NMA	Large
SpiNNaker 2	UoM	ANNs/SNNs	Training	NMA	Large
TrueNorth (Akopyan et al., 2015)	IBM	SNNs	Inference	NMA	Large
Darwin (Shen et al., 2016)	ZJU	SNNs	Inference	NMA	Small
Darwin II (Ma et al., 2017)	ZJU	SNNs	Training	NMA	Large
Darwin III (Ma et al., 2024)	ZJU	SNNs	Training	NMA	Large
DeepSouth (Wang et al., 2017)	Westwell	SNNs	Inference	NMA	Large
Intel SNN chip (Chen et al., 2018)	Intel	SNNs	Training	NMA	Large
ODIN (Frenkel et al., 2018)	K.U.Leuven	SNNs	Training	NMA	Small
Loihi (Davies et al., 2018)	Intel	SNNs	Training	NMA	Large
Tianjic (Pei et al., 2019)	Tsinghua	ANNs/SNNs	Training	NMA	Large
MorphIC (Frenkel et al., 2019)	UZH	SNNs	Training	NMA	Small
Flash-based (Wu et al., 2020)	–	ANNs/SNNs	Inference	CIM	Small
Loihi II (Davies, 2021)	Intel	SNNs	Training	NMA	Large
Y. Kuang et al. (Kuang et al., 2021)	PKU	ANNs/SNNs	Inference	NMA	Large
H2Learn (Liang et al., 2021)	UCSB	SNNs	Training	SNN TA	Large
SATA (Yin et al., 2022)	Yale	SNNs	Training	SNN TA	Small

SNN TA, SNN training accelerator; Arch, architecture.

Inspired by the simultaneous computation and storage capabilities of the brain's neural system, brain-inspired chips often adopt near-memory (NMA) or compute-in-memory architectures (CIM), incorporating closely coupled computational and storage resources within each computing core (Akopyan et al., 2015; Pei et al., 2019). Efficient intra-chip and inter-chip interconnects enable large-scale computational parallelism and high local memory, reducing computational power consumption.

The near-memory computing architecture refers to the separation of memory storage and computation in each processing unit, but with proximity. Key chips in this category include IBM's TrueNorth (Akopyan et al., 2015), Intel's Loihi (Davies et al., 2018; Davies, 2021), the University of Manchester's SpiNNaker (Painkras et al., 2013), Stanford University's Neurogrid (Benjamin et al., 2014), Heidelberg University's BrainScaleS (Schemmel et al., 2010; Pehle et al., 2022), Tsinghua University's Tianji Chip (Pei et al., 2019), and Zhejiang University's Darwin Chip (Shen et al., 2016; Ma et al., 2017, 2024). They utilize characteristics of brain-like spiking computation such as sparsity, spike summation, and asynchronous event-driven processing to achieve ultra-low power consumption, currently mainly supporting model inference and local online learning based on STDP, Hebb, etc. For instance, ROLLS (Qiao et al., 2015), ODIN (Frenkel et al., 2018), and MorphIC (Frenkel et al., 2019) support spike-driven synaptic plasticity (SDSP) rules, and Loihi adds a learning module for STDP rules. In SpiNNaker (Painkras et al., 2013) and BrainScaleS (Schemmel et al., 2010), STDP learning is exhibited through timestamp recording and learning circuits. In their next generations (Pehle et al., 2022), more flexible learning rules are possible due to the presence of embedded programmable units. Tsinghua University's Tianji Chip, as the first chip to support the fusion of SNN and ANN computation, improves accuracy based on ANN, and achieves rich dynamics, high efficiency, and robustness based on SNN. This mode is also adopted by BrainScaleS-2 (Pehle et al., 2022), SpiNNaker-2, and Loihi-2 (Davies, 2021). Recently, BPTT has been applied to SNNs, achieving higher accuracy compared to local learning rules (Wu et al., 2018, 2019). Some works, like H2Learn (Liang et al., 2021) and SATA (Yin et al., 2022), have designed specific architectures for BPTT learning in SNNs. In the future, the integration of learning rules will become increasingly important for exploring large and complex neuromorphic models in brain-inspired computing (BIC) chips.

Another important type of BIC architecture is the compute-in-memory architecture, where in-core processing units and on-chip storage are physically integrated, performing synaptic integration matrix operations in synaptic memory. Compute-in-memory chips can be divided into two categories based on the materials: traditional or emerging memories. Traditional memories (such as SRAM, DRAM, and Flash) can be redesigned to support specific logical operations (Wu et al., 2020). Their advantages include a mature ecosystem, easy simulation, and manufacturing. Emerging memories mainly refer to storage devices based on memristors. Synaptic weight storage, multiplication calculations, and presynaptic inputs are performed at the same crosspoint in the memristor, integrating computation and storage. Brain-inspired computing hardware based on memristors involves multiple levels of material and architectural designs, which is currently still in a small-scale phase due to manufacturing process limitations.

Multiple types of brain-like chips have shown remarkable developments, demonstrating significant advantages in terms of biological simulation and low-power inference. However, they still face numerous challenges in practical applications. When it comes to handling high-level intelligence tasks, the superiority of brain-like chips compared to GPUs and ANN accelerators has not been fully established. Currently, to optimize their performance, some designs draw inspiration from ANN accelerators for improvements. It's worth noting that current brain-like chips do not yet support the training of large-scale SNNs and require special architectural designs to accommodate the training process for SNNs. To further support large-scale SNNs, it is necessary to enhance brain-like systems from both a software and hardware perspective in a more collaborative manner.

### 5.3 Software and hardware interplay

The deployment of algorithms for SNNs onto neuromorphic chips typically requires certain software frameworks. The computational software frameworks mentioned in Section 5.1 usually support simulations on mainstream CPUs and GPUs, without clear mention of deployment on neuromorphic chips. Meanwhile, among the previously mentioned neuromorphic chips in Section 5.2, only about 27% of them are connected to application software packages (Schuman et al., 2022). Typically, these application software packages contain model construction tools, simulators, and optimization tools. Model construction tools are used to define the structure and parameters of neural networks, including neuron types, connection patterns. Simulators are applied to simulate and debug neural network models on the chip. Optimization tools are adopted to train the network parameters and optimize its performance. Here are some typical examples. The Neurogrid chip is paired with the Neurogrid Software Framework (Benjamin et al., 2014), allowing users to specify neuronal models in the Python programming environment. The software framework for the BrainScaleS chip is the BrainScaleS-Software-Stack (Pehle et al., 2022), which supports training neural networks on the chip using the PyTorch framework. The IBM TrueNorth chip typically utilizes a software framework called the TrueNorth Ecosystem, which is developed by the TrueNorth native Corelet language (Akopyan et al., 2015). IBM NorthPole (Modha et al., 2023) is a brain-inspired memory-near-compute chip with a software development kit, but this chip does not emulate spiking communication. Tianjic's software toolchain supports both ANN-to-SNN conversion and direct training for SNNs, and supports automatically transforming a pretrained model into an equivalent network that meets the Tianjic hardware constraints for non-spiking ANNs (Pei et al., 2019). The latest Darwin3 builds a specialized instruction set architecture (ISA) (Ma et al., 2024), which is close to machine code tailored for efficient neuromorphic computing. These software frameworks enable users to conveniently construct, simulate, and optimize neural network models on neuromorphic chips, facilitating efficient research and application development.

## 6 Applications of deep spiking neural networks

SNNs offer powerful computation capability due to their event-driven nature and temporal processing property. Theoretically, SNNs could be applied to any field where conventional deep neural networks (DNNs) are applied. As the training methods and programming frameworks of deep SNNs become more powerful, deep SNNs are increasingly drawing more attention and being applied to more fields, mainly including computer vision, reinforcement learning and autonomous robotics, biological visual system modeling, biological signal processing, natural language processing, equipment safety monitoring, and so on. It should be noted that this paper only lists some typical examples in recent years for some common application fields, not aiming to fully review all related studies.

### 6.1 Applications in computer vision

As traditional DNNs, the most common applications of SNNs lay in computer vision tasks. There are mainly two types of visual inputs for SNNs, i.e., RGB frames from traditional cameras or events from neuromorphic vision sensors. Neuromorphic vision sensors display great potential for computer vision tasks under high-speed and low-light conditions (Li and Tian, 2021). SNNs are excellent candidates for processing neuromorphic signals due to their event-driven nature and energy-efficient computing.

Recognition task plays an important role in the rapid progress of deep SNNs. SNNs are usually tested on both static datasets such as CIFAR10, CIFAR100, ImageNet, and neuromorphic datasets such as CIFAR10-DVS and DVS128-Gesture. Table 2 lists the performances of some recently proposed architectures. Besides common recognition tasks, deep SNNs are increasingly applied to more computer vision tasks, including object detection/tracking, image denoising/generation, image/video reconstruction, video action recognition, image segmentation, and so on.

#### 6.1.1 Object detection and object tracking

The first spike-based object detection model Spiking-YOLO was obtained through the ANN-to-SNN conversion method, achieving comparable performances to tiny-YOLO on PASCAL VOC and MS-COCO dataset with 3,500 time steps (Kim et al., 2020). Later, a spike calibration (SpiCalib) method was proposed to reduce the time steps to hundreds (Li et al., 2022). Kugele et al. (2021) and Cordone et al. (2022) combined some spiking backbones with an SSD detection head for event cameras.

Considering that the Siamese networks have achieved remarkable performances in object tracking, SiamSNN was constructed by conversion to achieve short latency and low precision degradation on several benchmarks (Luo et al., 2022). Similarly, the directly trained Spiking SiamFC++ showed a small precision loss compared to the original SiamFC++ (Xiang et al., 2022). A spiking transformer network called STNet was developed for event-based single-object tracking, demonstrating competitive tracking accuracy and speed on three event-based datasets (Zhang et al., 2022a). To process frames and events simultaneously, Yang et al. (2019) proposed DashNet, achieving good tracking performance with a surprising tracking speed of 2,083 FPS on neuromorphic chips.

#### 6.1.2 Image generation/denoising and image/video reconstruction

Generation tasks are increasingly explored in SNNs. Comşa et al. (2021) introduced a directly trained spiking autoencoder to reconstruct images with high fidelity on MNIST and FMNIST datasets. Kamata et al. (2022) constructed a fully spiking variational autoencoder (FSVAE), generating images with competitive quality compared to conventional ANNs. Liu et al. (2023) proposed a Spiking-Diffusion model, outperforming the existing SNN-based generation models on several datasets. Castagnetti et al. (2023) developed an image denoising solution based on a directly trained spiking autoencoder, achieving a competitive signal-to-noise ratio on the Set12 dataset with significantly lower energy.

Visual information reconstruction is important for neuromorphic vision sensors, because humans cannot directly perceive visual scenes from events. Zhu and Tian (2023) provided a comprehensive review of visual reconstruction methods for events. Duwek et al. (2021) proposed a hybrid ANN-SNN model, accomplishing image reconstruction for simple scenes from N-MNIST and N-Caltech101 datasets. Zhu L. et al. (2021) proposed an image reconstruction algorithm that combines DVS and Vidar signals, leveraging the high dynamic range of DVS to improve reconstruction effectiveness. Subsequently, they developed a deep SNN with an encoder-decoder structure for event-based video reconstruction, achieving performance comparable to ANN counterparts with only 0.05x energy consumption (Zhu L. et al., 2022).

#### 6.1.3 Others

Besides the above-mentioned tasks, deep SNNs are also applied in some other computer vision tasks, including video action recognition (Panda and Srinivasa, 2018; Wang et al., 2019; Zhang et al., 2022c; Chakraborty and Mukhopadhyay, 2023; Yu et al., 2024), image segmentation (Parameshwara et al., 2021; Kim et al., 2022; Liang et al., 2022; Zhang H. et al., 2023), optical flow estimation (Lee et al., 2020a; Cuadrado et al., 2023; Kosta and Roy, 2023), depth prediction (Rançon et al., 2022; Wu et al., 2022; Zhang et al., 2022b), point clouds processing (Zhou et al., 2020; Ren D. et al., 2023), human pose tracking (Zou et al., 2023), lip-reading (Bulzomi et al., 2023), emotion/expression recognition (Wang B. et al., 2022; Barchid et al., 2023), medical image classification (Shan et al., 2022; Qasim Gilani et al., 2023), and so on.

### 6.2 Applications in other fields

Besides computer vision tasks, SNNs are showing gradually expanding application prospects in many fields, including reinforcement learning and autonomous robotics, biological visual system modeling, biological signal processing, natural language processing, equipment safety monitoring, and so on.

#### 6.2.1 Reinforcement learning and autonomous robotics

As reinforcement learning (RL) is critical for the survival of humans and animals, there is increasing interest in applying brain-inspired SNNs to reinforcement learning. To reduce the latency of spiking RL, Qin et al. (2023) applied learnable matrix multiplication to encode and decode spikes.

Due to the good biological plausibility and high energy efficiency, SNNs have been applied to autonomous robotics for a long time, which is still a flourishing research direction, mainly including pattern generation (walk, trot, or run), motor control, and navigation (simultaneous localization and mapping, SLAM). Yamazaki et al. (2022) have already provided a good review of relevant studies, we do not go into more detail about this topic in this review which mainly focuses on deep SNNs.

#### 6.2.2 Biological visual system modeling and biological signal processing

ANNs play important roles in modeling biological visual pathways. However, SNNs are more biologically plausible models due to the use of temporal spike sequences. Therefore, several studies adopted SNNs to model the biological visual cortex. Further, they added a brain-inspired recurrent module into deep SNNs, outperforming the forward deep SNNs under natural movie stimuli (Huang et al., 2023b). Zhang J. et al. (2023) compared performances of deep SNNs and CNNs in the prediction of visual responses to naturalistic stimuli in three brain areas. Ma et al. (2023) presented a temporal conditioning spiking latent variable model to produce more realistic spike activities.

Due to the intrinsic dynamics, SNNs are also applied to process biological signals. Xiong et al. (2021) proposed a convolutional SNN for odor recognition of electronic noses.

#### 6.2.3 Others

SNNs were also applied to natural language processing, equipment safety monitoring, semantic communication, multi-modal information processing, and so on. To ease the heavy energy cost of ANN-based large language models, some studies applied SNN-based architectures, including SpikBERT (Lv et al., 2023), SpikingBERT (Bal and Sengupta, 2024), SpikeGPT (Zhu et al., 2023), and SpikeLM (Xing et al., 2024). Applications regarding equipment safety monitoring mainly include battery health monitoring (Wang et al., 2023a,b), autonomous vehicle sensors fault diagnosis (Wang and Li, 2023), and bearing fault diagnosis (Xu et al., 2022). Applications in semantic communication mainly tried to mitigate the limitation of transmission bandwidth (Wang M. et al., 2023). Applications in multi-modal information processing currently show up in audio-visual zero-shot learning tasks (Li et al., 2023a,b).

### 6.3 Discussion on SNN applications

Deep SNNs have achieved great success in many fields in recent years, but there still exist some limits that need to be addressed. Firstly, although many studies demonstrated that deep SNNs achieved comparable accuracy to their ANN counterparts on many tasks, they still lag behind conventional ANN SOTA, especially for large datasets like ImageNet, which asks for more endeavors. Secondly, many studies claimed that the proposed SNNs consumed much less energy compared to ANN counterparts, through calculating the number of addition operations, without considering the cost of other operations like data movement. Therefore, it is meaningful to deploy well-performed SNNs on neuromorphic chips or corresponding simulators to fully exploit the event-driven nature and measure the actual energy cost. Thirdly, as for applications requiring high processing speed and low power consumption, like robotics, it is promising to adopt neuromorphic vision/audio sensors and neuromorphic processing chips due to their event-driven nature, besides network pruning and weight quantization. Meanwhile, to fully exploit the advantages of events, it deserves more efforts to explore how to directly process neuromorphic sensing events using SNNs, without converting events into frames as current studies usually do. Fourthly, as for transformer-based SNNs used in language or video processing, how to choose the input clip for one simulation step, to reconcile the temporal resolution of the input sequence and the simulation step of SNNs, is worth studying. Last but not least, as SNNs have an additional temporal dimension, how to achieve the speed-accuracy trade-off as humans is a problem worth of study. In other words, how to assign a suitable simulation duration or how to decide when to make a choice, are important questions to realize the balance between computation cost and prediction accuracy.

## 7 Future trends and conclusions

This article provides an overview of the current developments in various theories and methods of deep SNNs, including relevant fundamentals, various spiking neuron models, advanced models, and architectures, booming software tools and hardware platforms, as well as applications in various fields. However, there are still many limitations and challenges.

(1) Currently, only a few aspects of the intelligent brains have been applied to instruct the construction and training of SNNs, lacking enough biological plausibility. Therefore, to improve SNNs' capability, it is necessary to introduce more types of spiking neurons, rich connection structures, multiscale local-global-cooperative learning rules, system homeostasis, etc., into SNNs to more accurately mimic the cognitive and intelligent characteristics emerging in the brains. For example, it deserves more efforts to train SNNs with self-supervised or unsupervised learning (Zhou Z. et al., 2024), as children mainly receive unlabeled data during growth. Besides, the brain is actually a complex network, thus it is worthy of more effort to study graph SNNs, although some attempts already exist (Li et al., 2024; Yin et al., 2024).

(2) Recent neuroscience studies have found that astrocytes can naturally realize Transformer operations (Kozachkov et al., 2023), which provides a new direction for the improvement of SNNs. In addition, astrocytes have the function of regulating neuronal firing activity and synaptic pruning (Lee et al., 2021; Liu et al., 2022), which provides ideas for the performance improvement and lightweight of SNNs in the future.

(3) Information encoding methods and training algorithms for SNNs are mostly based on average firing rates, lacking the ability to represent temporal dynamics adequately. There should be more exploration of time-dependent information encoding strategies and corresponding training algorithms, to further enhance the spatiotemporal dynamic characteristics of SNNs and strengthen their temporal processing capability.

(4) The training of SNNs mainly employs time-dependent methods, like BPTT, which greatly increases the training cost, compared to conventional DNNs. Thus, there is a need to develop brain-like SNNs that can be trained in parallel, and dedicated software and hardware that support their computation, reducing training time and power consumption.

(5) As there are obstacles to conversion and interaction between different neuromorphic platforms, it is needed to establish a common standard to improve interoperability. Further, more brain-inspired principles or technologies should be incorporated into the design of neuromorphic systems, to enhance the computational performance of the chips, in terms of processing speed and energy efficiency.

(6) Large-scale SNNs are mainly applied to classification tasks. Their potential in handling tasks that need to process continuous input streams, such as videos, languages, events from neuromorphic vision sensors, etc., has not been fully explored. Moreover, the introduction of various neuromorphic sensors and neuromorphic chips into autonomous robotics, cooperating with conventional sensors and processing chips, might be an efficient and effective way to achieve embodied intelligence. Further studies are needed to fully leverage the features and advantages of SNNs.

In summary, studies and applications of SNNs are growing rapidly, but there is still great potential to improve the effectiveness and efficiency of SNNs. Efforts should be made in multiple directions, including model architectures, training algorithms, software frameworks, and hardware platforms, to promote the coordinated progress of models, software, and hardware.

## Author contributions

CZ: Writing – original draft, Writing – review & editing. HZha: Writing – original draft, Writing – review & editing. LY: Writing – original draft, Writing – review & editing. YY: Writing – original draft, Writing – review & editing. ZZ: Writing – review & editing. LH: Writing – review & editing. ZM: Funding acquisition, Project administration, Resources, Supervision, Writing – review & editing. XF: Writing – review & editing, Resources. HZho: Writing – review & editing, Resources. YT: Writing – review & editing, Resources.
